# The Influences of RARγ on the Behavior of Normal and Cancer Stem Cells

**DOI:** 10.3390/ijms27031291

**Published:** 2026-01-28

**Authors:** Geoffrey Brown

**Affiliations:** Department of Biomedical Sciences, College of Medicine and Health, University of Birmingham, Edgbaston, Birmingham B15 2TT, UK; g.brown@bham.ac.uk; Tel.: +44-(0)121-414-4082

**Keywords:** retinoic acid receptors, normal stem cells, cancer stem cells, embryogenesis, hematopoiesis

## Abstract

Retinoic acid receptor (RARγ) mRNA is expressed spatially and temporally during mouse embryogenesis and largely within stem and progenitor cells, indicating a role in organ formation. RARγ agonism promoted the maintenance of hematopoietic stem cells, and blocked stem cell development as shown for hematopoiesis, zebrafish development, and chondrogenesis. Transgene expression enhanced the generation of induced pluripotent stem cells, indicating a role in ground-state pluripotency. RARγ is oncogenic in acute myeloid leukemia, cholangiocarcinoma, and colorectal, head and neck, hepatocellular, ovarian, pancreatic, prostate, and renal cancers. RARγ agonism or overexpression enhanced the proliferation of cancer cells. Conversely, antagonism or inhibition of all-trans retinoic acid synthesis led to the death of cancer cells including cancer stem cells. The pathways regulated by RARγ, via canonical activation and repression of gene expression, include Wnt/β-catenin and Notch signaling. RARγ also acts as a co-factor to Smad3 and reduced or enhanced TGFβ-driven and Smad3-mediated events when liganded and non-liganded, respectively. Collectively the findings support the view that RARγ plays a crucial role in controlling stem and progenitor cell behavior.

## 1. Introduction

The cancer stem cell (CSC) concept has focused considerable attention on the nature of normal stem cells and how they regulate their behavior. The concept revised our understanding of cancer in stating that CSCs, like normal stem cells, generate a hierarchy of cells [1]. In 1997, Dominique Bonnet and John Dick described a rare population of acute myeloid leukemia (AML) patients’ cells that initiated human AML when transplanted into non-obese diabetic mice with severe combined immunodeficiency. The leukemia-initiating cells had self-renewed as expected of a leukemic stem cell (LSC). They were CD34^++^ CD38^−^, like hematopoietic stem cells (HSCs), and differentiated in vitro into leukemic blasts. Therefore, AML arises from transformation of an HSC whereby LSCs sustain a patient’s highly proliferative myeloblasts. CSCs have been identified for many cancers as cells that self-renew, proliferate, and generate offspring leading to cancer [2]. Whether there is a difference between normal stem cells and CSCs that can be targeted is central to developing better treatments for cancer. CSCs are quiescent, like some HSCs. Chronic myeloid leukemia arises from an HSC and chemotherapy, which targets dividing cells, failed to eliminate CML CSCs [3]. The tyrosine kinase inhibitor imatinib is used to treat CML and LSCs were insensitive to this agent when treated in vitro, despite inhibition of the tyrosine kinase activity of *BCR-ABL* fusion oncogene [4,5]. CML LSCs cause disease relapses as shown from allogeneic transplantation studies [6] and there is good support for the view that CSCs play a key role in cancer metastasis, reviewed in [7].

One starting point to examine the differences between normal stem cells and CSCs is to focus on molecules that play a crucial role in controlling normal stem cell behavior. Profiling of the expression of mRNAs for members of the steroid hormone nuclear receptor family (NRs) within undifferentiated human and mouse embryonic stem cells (ESCs) identified the presence of 42 mRNAs for NRs. Twenty-nine were expressed by human and mouse ESCs and RARγ mRNA was shared by human H1 and H9 ESCs and mouse CMT1-1 ESCs. Mouse ESCs expressed RARγ mRNA at a level that was >10-fold higher than any other NR. On day 1, there was a marked decline in the expression of RARγ mRNA as human and mouse ESCs differentiated towards embryoid bodies. Hence, RARγ plays a core role in controlling the behavior of undifferentiated ESCs [8].

RARs are abnormally expressed in cancer. The PML-RARα fusion protein is the master driver of acute promyelocytic leukemia [9,10] and provided a paradigm for an oncogene-targeted cure. It interferes with the expression of genes that regulate normal stem/progenitor cell behavior in leading to a global downregulation of 26 *Hox* genes from a comparison of patients’ bone marrow cells to normal controls [11]. Surprisingly, when artificial PML-RAR fusion proteins were generated for the RARα, β, and γ isoforms, they all had an oncogenic potential from in vitro studies [12]. Moreover, the *RARG* gene is rearranged instead of the *RARA* gene in some patients with AML resembling acute promyelocytic leukemia giving rise to PML-RARγ and fusion proteins from other RARγ translocations [13]. The *RARG* fusion proteins promoted HSC/progenitor cell self-renewal by upregulating BCL2 and ATF3 [14] and essentially an adaptive response to cellular stresses. The most prevalent fusion protein CPSF6-RARγ interacted with histone deacetylase 3 to promote transformation and suppressed gene expression that is associated with myeloid differentiation. Synergy between CPSF6-RARγ and RAS mutations was proposed to drive aggressive AML [15]. RARγ is oncogenic in cholangiocarcinoma and colorectal, head and neck, hepatocellular, ovarian, pancreatic, prostate, and renal cancers. Overexpression has been linked to enhanced cell proliferation, rapid tumor progression, and a poor disease prognosis, reviewed in [16]. Cancer is viewed as a genetic developmental disorder [17] but environmental factors are important [18]. Even modest perturbation of the availability of ATRA to cells leads to developmental abnormalities [19], and whether perturbations dysregulate stem cell biology leading to cancer is largely unexplored.

The models used to study the roles of RARs range from whole animals and embryos to the use of cell lines. This review examines the various findings regarding how RARγ influences the behavior of normal stem cells and CSCs.

## 2. Some Principles on the Roles of RARs and ATRA

The cells of jawed vertebrates express three main types of RARs, namely RARα, β, and γ and there are multiple splice variants of each. The well-established and canonical role of RARs is to regulate gene expression as a ligand-activated transcription factor. The three receptors bind ATRA, the most active metabolite of vitamin A, with a high affinity to drive gene expression. To do so, RARs form obligatory and various heterodimers with the three retinoid X receptors RXRα, β, and γ. Heterodimers are bound to the retinoid response elements (RAREs) in the promoter regions of ATRA target genes. The RARs need the assistance of regulators to modulate transcription. In the absence of ATRA, corepressors are recruited, leading to suppression of gene expression. The presence of ATRA leads to the release of corepressors, the recruitment of coactivators, and gene expression [20].

Hence, the availability of ATRA to cells is an important consideration in interpreting the findings from RAR studies that make use of cell line cells. They are generally grown in medium plus 10% fetal calf serum which contains 50 nM all-trans retinol and ~0.4 to 1.4 nM all-trans retinoic acid (ATRA) [21]. This level of ATRA is significant because sub-nM is sufficient to transactivate RARγ and RARβ (IC_50_ = 0.36 and 0.39 nM for CV-1 cells, respectively) whereas thirty-five-fold more ATRA is required to transactivate RARα (IC_50_ = 12.8 nM) [22,23]. That unliganded RARγ can mediate a level of gene transcription has also been proposed [24]. Therefore, RARγ is highly likely to be active within serum-grown cells whereby at least RARγ2 self-regulates its synthesis through a RARE that is embedded in Sp1 sites [25]. The presence of all-trans retinol in serum is also a consideration for studies of RARs because cell line cells are heterogeneous and some of the cells may use to synthesize ATRA. Whether sufficient ATRA is made by cells to activate RARα effectively is uncertain. Notwithstanding, the presence of precursors to ATRA biosynthesis is highly relevant to concluding whether RARs and ATRA do or do not play a role, as seen for mouse embryonic stem cells (ESCs). They were cultured in medium plus the B27 supplement, which contains retinyl acetate for ATRA synthesis, and differentiated towards Sox 1^+^ neural progenitors. ATRA was not measurable within the ESCs. However, the generation of neural progenitors was reliant on ATRA produced endogenously because their formation was inhibited by either removing retinyl acetate from the supplement, inhibiting the enzymes for ATRA biosynthesis, or the use of the pan-RAR antagonist AGN193109. Neuroectodermal differentiation was restored by the addition of just 1 nM ATRA to the retinyl acetate-deprived cells [26]. Sub-nM levels of ATRA are biologically active in cell culture experiments.

A longstanding question is, do the main RAR isoforms play unique roles to regulate cell development? To examine this question regarding ATRA target genes, individual receptors were knocked out in cell lines. From in vitro studies of the differentiation of F9 and P19 embryonal carcinoma cells and having knockout of individual receptors, there was some redundancy within RARs. Isoforms functionally substituted each other regarding the induction of target genes and cell differentiation. Either RARα or RARγ mediated ATRA-induced differentiation of P19 cells into various types of cells. However, an isoform can play a unique role and/or override the activity of other RARs in a manner that is cell context-specific. Only RARγ mediated ATRA-induced differentiation of wild-type F9 cells into a primitive endoderm [27]. Even so, knockout studies are confounded because receptor absence is not the same as the receptor being present and inactive and we do not yet understand the importance of inactive RARs. A means to resolve this is the use of antagonists of all RARs, RARα, and RARγ, and highly selective RARβ antagonists remain elusive. These are synthetic compounds and, therefore, pharmacological tools. Regarding selective agonists, the retinoid structure has been modified substantially to allow selective binding to an individual RAR. Antagonists reverse the transactivation action of ATRA by displacing ATRA and not allowing transactivation in reporter assays. Again, the retinoid structure has been modified to obtain antagonists that selectively bind to an individual RAR. There is no evidence to support the existence in nature of agonists and antagonists that are selective for an individual RAR. Cells that have been adapted to grow in serum-free conditions (ITS^+^ serum replacement) are beneficial to a more precise control on antagonist silencing versus ATRA activation of RARs. For serum-free-grown cells, RARγ is still likely to be active because of ATRA carryover by bovine serum albumen, a component of the ITS+ supplement (at 0.125 g/mL). ATRA binds to serum albumin, via hydrophilic and hydrophobic interactions, and is transported by albumin to allow a controlled uptake by cells [28,29]. RARα is highly likely to be inactive within serum-free-grown cells because of a lack of any biosynthesis from serum all-trans retinol.

The use of ATRA and the synthetic RAR selective agonists and antagonists to mimic normal physiological conditions is central to unraveling the physiology of RARs. Normal tissue cells encounter ∼1–10 nM ATRA [30] and accordingly ATRA binds to RARα, RARβ, and RARγ with nM affinities. The ED_50_ values were 3–10 nM from studies that used crude protein extracts of RARα, β, and γ, and unlabeled ATRA and ^3^H ATRA for competition binding studies [23]. The values are close to the EC_50_ values from transactivation studies other than RARβ and RARγ are transactivated by sub-nM ATRA (as above). Potent and highly selective RARα and RARγ agonists and antagonists are available commercially and the compounds have nM ED_50s_ for RARs, like ATRA, and are highly selective [31]. Antagonists are potent in blocking ATRA transactivation of RARs. The use of 10^−7^ M ATRA is sufficient to drive neutrophil differentiation of the promyeloid cell line HL-60 when grown in serum [32,33] and is appropriate because the cells have genomic mutations involving oncogenes [34]. The co-addition of 10^−7^ M of either the pan-RAR antagonist AGN194310 or the RARα selective antagonist AGN194310 blocked ATRA-induced neutrophil differentiation. In contrast, many developmental studies make use of ATRA at 5 × 10^−6^ M, and at even higher levels. For example, 5 × 10^−6^ M ATRA was used for neuronal differentiation of E14Tg2A mouse ESC [35] and embryoid bodies [36]. Five thousand-fold more than the ~1 nM ATRA for some tissues is considered pharmacological rather than a physiological amount, particularly as low-level gradients guide embryogenesis [37]. To mimic normal physiological conditions, ATRA is used to treat cells in vitro from 1 to 100 nM, and 10 × 10^−6^ M is entering the realm of toxicity studies. The toxic effects of ATRA and synthetic retinoids include changes to lipids and membranes [38]. Toxicity is more of a concern regarding synthetic analogs that have complex ring structures which, when used at 10 × 10^−6^ M, induce DNA damage without altering viability [39]. The excessive use of ATRA and synthetic retinoids also leads to unwanted perturbations to developmental processes and pathways because retinoids are teratogenic [40].

## 3. RARγ Agonism Blocks the Development of Stem/Progenitor Cells

ATRA activation of RARs is crucial to mouse embryogenesis. Embryos died in utero from vitamin A deficiency [41] and double RAR knockout embryos invariably died either in utero or shortly after birth [42]. RARγ mRNA is expressed spatially and temporally during mouse embryogenesis, indicating roles in early morphogenesis, chondrogenesis, and squamous epithelial differentiation. mRNA was detected when mesoderm developed from the primitive streak (day 6 post conception) and in all germ cell layers in the posterior embryo region (day 8). High levels were found in frontonasal and pharyngeal mesenchymal derivatives (day 12.5), neural ectoderm and endoderm derivatives and when epithelia had started to differentiate (day 13.5), the root cells of developing whisker follicles, and where teeth develop [43]. Zebrafish express the paralogs RARγa and RARγb. During embryogenesis, the three streams of migrating cranial neural crest cells expressed RARγ mRNA, and expression was high in the anterior and tailbud regions [44]. Other workers have reported restriction of RARγ mRNA expression to mesodermal and neural crest stem/progenitor cells in the head area, the lateral plate mesoderm, and the presomitic mesoderm of the tail bud [45]. These findings support the view that RARγ plays a decisive role within stem and progenitor cells.

RARγ is the predominant RAR that is expressed in the skin of humans and mice and is expressed constitutively by cultured human keratinocytes, and dermal fibroblasts [46]. The mRNA expression was localized to all epidermis layers, the outer root sheath of hair follicles, follicular hair bulbs, and eccrine and sebaceous glands [47]. The topical application of ATRA and the RARγ agonist BMS189961 to the skin of mice led to pronounced epidermal proliferation and increased the expression of ATRA target genes. Treatment with the RARα agonist BMS753 strongly decreased the expression of target genes, indicating different roles for RARα and RARγ in regulating skin homeostasis. Other findings have led investigators to suggest that dysregulated RAR signaling plays a role in skin diseases [48].

**Figure 1:**
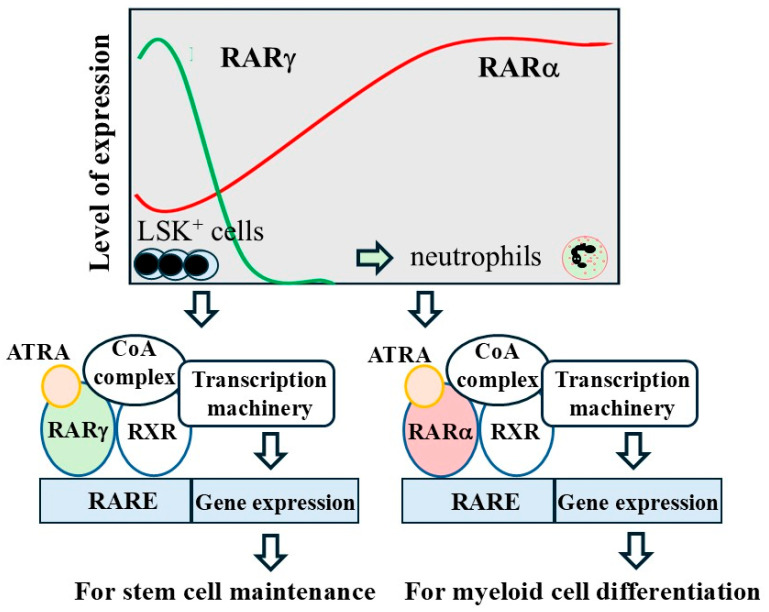
RARγ and RARα maintain HSCs and facilitate myeloid differentiation, respectively. The upper panel shows the changes to the levels of expression of RARγ and RARα during myelopoiesis and below that active RARγ and RARα play roles in stem cell maintenance and myeloid cell differentiation, respectively. CoA, coactivator.

Expression of RARs is developmentally regulated during adult hematopoiesis. Mouse lineage^−^, c-kit^+^, and Sca-1^+^ (LKS^+^) cells contained HSCs whereas lineage^−^, c-kit^−^, and Sca-1^−^ (LKS^−^) cells did not. LSK^+^ cells selectively expressed RARγ1 and RARβ2 mRNA, and RARα mRNA was expressed by both LKS^+^ and LKS^−^ cells [49]. It is well known that liganded RARα facilitates myeloid differentiation [50]. Expression of RARα2 was increased dramatically when the factor-dependent cell-Paterson (FDCP)-mixA4 murine progenitor cells were induced to undergo myelomonocytic differentiation [51]. Regarding RARγ, there was a 3.3-fold reduction in the number of HSCs with a profound decrease in the very primitive and multilineage repopulating cells within the bone marrow of femurs of RARγ^−/−^ mice. This abnormality was not seen for RARα^−/−^ mice. Progenitor cells had increased in the femurs of RARγ^−/−^ mice, indicating enhanced differentiation of HSCs into a mature cell compartment. RARγ needs to be active to maintain HSCs. As measured by transplantation studies, ATRA promoted the maintenance of HSCs in vitro, and this potentiating effect was abrogated by loss of RARγ. Similarly, when LSK^+^ cells from RARγ^+/+^ and RARγ^−/−^ mice were cultured without ATRA for 14 days they failed to show a competitive repopulating potential. Primitive hematopoietic precursors that had been transduced with RARγ1 exhibited a substantially more undifferentiated phenotype [49]. Conversely, an increase in mature myeloid cells was seen for conditional RARγ^−/−^ mice and when wild-type mice were treated with the pan-RAR antagonist AGN194310 [52]. From the above, active RARγ and RARα appear to ensure a proper balance to the conduct of hematopoiesis via their different roles in supporting stem cell maintenance and myeloid cell differentiation, respectively (Figure 1).

HSCs that are resistant to regenerative stress preserve HSCs to ensure blood cell production. These long-term reconstituting human HSCs were identified by virtue of responding differently to regeneration-mediated stress when transplanted into immunodeficient mice. CD112^low^ cells reconstituted hematopoiesis but there was a transient restraint/latency. CD112^high^ cells, with less cells in G_0_ of the cell cycle, responded rapidly to reconstitute hematopoiesis. The resistance of CD112^low^ cells to regenerative stress was attributed to an integrated stress response that was uniquely activated and mediated by the master transcription factor activating transcription factor 4, which was highly expressed in HSCs [53]. An activated integrated stress response was also a marker for primary AML LSCs [54]. ATRA regulates the response of cells to stress by virtue of inducing a biphasic expression of the pro-apoptotic C/EBP homologous transcription factor (CHOP) within HL-60 and NB4 cells. Expression was high at 1 h, absent at between 6 and 24 h, and high at 48 h and negative regulation of C/EBPε-mediated expression of the myeloid-specific gene lactoferrin was attributed to CHOP upregulation [55]. Loss of RARβ within hepatocytes elevated the integrated stress response [56]. Whether activity of RARγ maintains HSCs by influencing the integrated stress response remains to be seen. The response can lead to two outcomes: pro-survival or apoptosis [57].

Tissue patterning was disrupted when zebrafish embryos were treated at 4 h post fertilization with 10 to 80 nM of the RARγ agonist AGN20578. The experiments were performed in fish water whereby RARα is inactive due to the lack of exogenous ATRA. RARγ activation blocked the development of tissues that were derived from cranial neural crest and the formation of the most posterior somites. The fish exhibited severe truncation, a lack of pectoral fin growth, and disruption to the formation of cranial bones and anterior neural ganglia, and developed cardiac edema. The lateral plate mesodermal stem/progenitor cells to pectoral fin development (Tbx-5^+^ cells) were intact because the agonist’s action was reversed by washout, at 27 h post fertilization, and by changing the medium to medium that contained 10 nM of the RARγ antagonist AGN205728. A lack of caudal fin formation was associated with a loss of *hoxb13a* gene expression in the tail. The RARγ agonist blocked caudal fin regeneration when the fin was transected at 2 days post fertilization, and washout of the RARγ agonist or subsequent treatment with the RARγ antagonist increased caudal fin length [58]. RARγ agonism had either blocked the generation of progenitors that were needed to give rise to the tissues affected, or such cells had been rendered unresponsive to the signals for their development. Regarding the latter, collectively the above defects are like those which occurred in mutant zebrafish that are defective in fibroblast growth factors (fgf) and their receptors [59,60]. They include shortening of the embryo axis (fgf8a plus fgf24), craniofacial abnormalities (fgf8), loss of pectoral fins (fgf24, fgf10a, or fgf16), and heart defects (fgf8). Agonism of RARγ may have interfered with the ability of cells to respond to fgf or some other factor.

The formation of cartilaginous nodules was blocked when micro-mass cultures of mesenchymal embryo limb cells, from day 11.5 embryos, were treated with the RARγ agonist NRX204647 or ATRA at 3 to 30 nM. The RARγ agonist prevented the formation of ectopic bone masses in mice when mesenchymal stem cells were treated in vitro with the RARγ agonist and then injected into nude mice. Agonist-treated cells had lost their skeletogenic potential and were unresponsive to treatment in vitro with recombinant bone morphogenic protein-2 and the overall levels of Smad proteins and phosphorylation of Smad1, Smad5, and Smad8 were decreased. Studies to confirm that RARγ agonism reduced bone morphogenic protein signaling made use of ATD5 chondrogenic cells transfected with the signaling reporter plasmid Id1-Luc. Treatment of these cells with recombinant bone morphogenic protein-2 increased luciferase activity and this was counteracted by the co-addition of the RARγ agonist CD1530. The RARγ agonist also blocked heterotopic ossification in a transgenic model of fibrodysplasia ossificans progressiva. The investigators proposed that RARγ agonism had blocked the chondrogenic phase of heterotopic ossification [61].

RARγ2 is the major isoform that is expressed throughout the caudal axial progenitor domain of vertebrates. It becomes active in late-stage embryos when axial elongation is terminated, indicating a role for RARγ2 in this process. For Xenopus embryos, increased expression of posterior *Hox* genes and that of marker genes for presomitic mesoderm and the chordoneural hinge was seen when the embryos were treated with the RARγ-inverse agonist NRX205099 (for increased repression) or when a dominant-negative RARγ was expressed. These findings supported the view that the pool of caudal progenitor cells was maintained during elongation by inactive RARγ2. Inactive RARγ had maintained presomitic mesoderm cells, which are viewed as progenitors in giving rise to future somites and their derivatives. When the embryos were treated with the RARγ agonist NRX204647, the expression of caudal genes was reduced, and extension of the body axis was terminated prematurely. For near the determination front and when axial elongation nears completion, the investigators proposed that caudal domain RARγ2 becomes active, due to ATRA proximity, and drives apoptosis to terminate elongation when the progenitor pool becomes exhausted. The involvement of RARγ2 in all stages of axial elongation highlights that the roles of RARγ are complex with active and inactive RARγ regulating cell behaviors in a manner that is context dependent [62]. From studies of HSCs, zebrafish, and chondrogenesis, RARγ agonism blocked stem cell development and the maintenance of progenitors in Xenopus embryos by inactive RARγ may relate to primitive cells being allowed to differentiate towards a more mature compartment.

A consensus from all the above findings is that the presence of RARγ is important to the proper maintenance of stem cells and can block stem/progenitor cell development whereby there is the need for RARγ to be liganded. The action of RARγ was attributed to unresponsiveness of cells to bone morphogenic protein and changes to Smad protein levels from studies of chondrogenesis. However, the precise mechanism of how the chondrogenesis phase to bone formation was blocked is confounded because active and inactive RARγ play different roles during tissue development, as seen for Xenopus.

## 4. RARγ Agonism Enhances the Generation of Induced Pluripotent Stem Cells (iPSCs)

Transgene expression of the four transcription factors Oct4, Sox2, c-Myc, and Klf4 reprograms mouse and human somatic cells to iPSCs but only a small percentage of mouse embryonic fibroblasts were reprogrammed by prolonged expression. Additional transgene expressions of RARγ and the orphan nuclear receptor liver receptor homolog 1 (LRH-1) substantially increased the frequency of reprogramming and shortened the course to only 4 days. The reprogrammed cells resembled ground-state mouse ESCs. The use of a dominant-negative form of *RARA* impeded reprogramming. The combination of six factors also readily reprogrammed primary human neonatal and adult fibroblasts to iPSCs [63].

In the above studies, mouse iPSCs were cultured in 15% fetal bovine serum and the human iPSCs in knockout serum replacement medium containing lipid-rich albumin, indicating the need for RARγ to be active. A follow-on study showed that removing retinol acetate, the precursor to ATRA synthesis, from the medium supplement NZB27/LIF impeded the reprogramming of mouse embryonic fibroblasts into iPSCs. Removal substantially decreased reprogramming (seven-fold) regarding transgene expression of Oct4, Sox2, c-Myc, Klf4, RARγ, and LRH-1 and to a lesser extent (two- to three-fold) when just Oct4, Sox2, c-Myc, and Klf4 were expressed. The use of the RARγ agonist CD437 had a positive effect on reprogramming. Three- to four-fold more reprogrammed colonies were obtained when CD437 was used with either expression of Oct4, Sox2, c-Myc, and Klf4 or these four factors plus LRH-1. Reprogramming was increased two- to three-fold when Oct4, Sox2, c-Myc, and Klf4 were expressed constitutively with expression of RARγ controlled in a Dox-inducible manner and post-induction of expression. RARγ failed to enhance reprogramming when retinol acetate was removed from the medium. Expression of the above four factors with just LRH-1 enhanced reprogramming, which was also not seen in the absence of retinol acetate [64]. Rapid reprogramming requires RARγ to be active and the action of LRH-1 is also dependent on the presence of liganded RARγ.

Transgene expression of LRH-1 was used to reprogram Oct4-GFP epiblast stem cells (EpiSCs) into iPSCs. Post recovery, the cells were switched to different media to examine the influence of retinoids. Reprogramming was substantially reduced by removing all-trans retinol from the N2B27 medium supplement. The addition of ATRA and particularly at 0.1 nM (sufficient to activate RARγ) to the all-trans retinol-free medium substantially increased colonies and a high concentration of the RARγ antagonist CD2665 (1 μM) had a deleterious effect. Surprisingly, RARγ overexpression reduced LRH-1-mediated reprogramming of EpiSCs. To examine whether RARγ interacted with the Wnt pathway and β-catenin within EpiSCs, complexes were pulled down using an antibody to RARγ and then probed with an antibody to β-catenin. Treatment of EpiSCs with the RARγ agonist CD437 led to substantial enrichment of β-catenin in the complex, indicating a ligand-dependent regulation of the availability of β-catenin. Conversely, there was a weak interaction between RARγ and β-catenin in the absence of CD437. TOPflash experiments, for reporting canonical Wnt signaling, examined crosstalk between the RAR and Wnt pathways. Chiron, a Wnt pathway activator, increased reporting and high concentrations had a deleterious effect on LRFH-1-mediated reprogramming of EpiSCs. TOPflash activity was significantly reduced by RARγ agonism (0.1 nM ATRA and CD437) but not by RARγ antagonism (CD2665). The investigators concluded that physical interaction between active RARγ and β-catenin had promoted EpiSCs reprogramming by modulating or negatively regulating Wnt signaling [64].

Wnts play a central role in regulating embryo axis formation, axon guidance, cell fate determination during organogenesis, and tissue modeling [65]. ATRA regulates the multiple Wnts and their receptors that are expressed by cells. When ATRA was used to induce neuronal differentiation of NT2/NTera2 cells that were derived from a human embryonal tumor, Wtns were downregulated (Wnts3A, 8A, 8B, and 10B) and upregulated (Wnts2, 7B, and 14B) and the receptors were upregulated (FZD4 and FZD10). A threshold model was proposed in which the effects of multiple Wnts are summed up regarding β-catenin actions to determine cell fate [66]. ATRA administration to mouse dams to expose embryos led to complete downregulation of β-catenin and LEF-1 within the early developing palate. The viable embryos exhibited cleft palate and severe limb abnormalities and the fetuses that died had a cleft palate. A negative influence of RARs on Wnt/β-catenin signaling appears to have altered cell fate, leading to the abnormalities to craniofacial tissues and limbs [67]. Crosstalk between the ATRA and Wnt signaling pathways leads to the development of heart cells from human iPSCs [68]. Combining signaling by 2.5 × 10^−7^ M ATRA and Wnt at a specific time induced human iPSCs to differentiate into sinus node-like cells. Increases in the proportion of iPSC-pacemaker cardiomyocytes and the expression of pace-related transcription factors and proteins were observed [69]. As seen for EpiESCs, RARγ modulation of Wnt signaling or a negative influence is highly relevant to naïve-like pluripotency.

RARγ activation and cooperativity with LRH-1 had a positive effect on the derivation of iPSCs from human dermal fibroblasts [70]. The iPSCs were made by transgene expression of a combination of Oct4, Sox2, Klf4, L-Myc, and p53, and the use of agonists of RARγ (CD437) and LRH-1 (RJW101) and inhibitors of GSK and MEK1/2. Transcripts of TGF-β superfamily members and TGF-β signaling pathway components were prominent within the iPSCs cells as seen from comparison of the transcriptome with published data. High levels of expression of TGF-β pathway-related molecules were confirmed by qtRT-PCR. When the iPSCs were cultured in the presence of the TGF-β signaling inhibitors SB431542 and A83-01, iPSCs declined as cells began to differentiate, suggesting a role for TGF-β in sustaining naïve-like pluripotency. TGF-β was able to substitute for the use of the agonists of RARγ and LRH-1 to induce naïve-like pluripotency and activation of TGF-β signaling was shown to be related to the gene transfection protocol plus the use of the agonists of RARγ and LHR-1. From these findings, enhanced TGF-β signaling is important to the generation and maintenance of iPSCs, as also mediated by RARγ activation and cooperativity with active LRH-1.

TGF-β signaling family members are master regulators of organogenesis in directing some of the earliest cell-fate decisions [71]. ATRA either suppresses or amplifies TGFβ signaling and, therefore, the action is context dependent [72]. TGF-β was required for the maintenance of ground-state pluripotency in human ESCs [73]. Blocking of TGF-β signaling regarding the conditions used to culture human ESCs caused the cells to differentiate [74]. They differentiated primarily down the neuroectoderm lineage when either TGF-β was removed from the medium or the TβR1 kinase inhibitor SB431542 was used to inactivate Smad2 and Smad3. Findings also revealed that Smad2 and Smad3 directly controlled the activity of the *Nanog* gene for pluripotency [75]. TGF-β signaling was also required to maintain the pluripotency of chemically reset human cR-H9 naïve pluripotent stem cells [76]. TGFβ is also a key player in cancer biology in modulating cell invasiveness and the microenvironment of cancer cells [77].

From findings for the generation of iPSCs, there is a consensus that agonism of RARγ plays a role in the molecular reprogramming of cells towards ground-state pluripotency. There was the need for liganded RARγ because the absence of all-trans retinol prevented the enhancement of the generation of IPSCs. The action of RARγ has been attributed to a negative influence on Wnt signaling, which can induce iPSCs to differentiate. RARγ enhancement of the generation of iPSCs also led to enhanced TGFβ signaling, as required for the maintenance of ground-state pluripotency, and TGFβ/activin have been used to establish human iPSCs [78]. How these events facilitate ground-state pluripotency is yet unclear. Even so, the need for liganded RARγ is in keeping with its importance for the maintenance of HSCs and that it can block stem/progenitor cell development.

The epigenetic landscape is important for the execution of stem cell fate [79]. That RARγ plays a role in modeling chromatin is supported by findings for RARγ knockout within mouse ESCs. For these cells, ATRA induced chromatin epigenetic marks and reorganization of the cluster of Hox genes. Deletion of the binding site for RARγ in the *Hoxa1* gene enhancer attenuated the transcriptional activation of the *Hoxa* and *Hoxb* clusters. ATRA activation of RARγ was necessary to erase the polycomb repressive mark H3K27me3 from activated Hox genes during ESC differentiation and, from the kinetics of epigenomic reorganization, complete erasure of the mark was not necessary to initiate *Hox* gene transcription. Reorganization of the *Hox* gene cluster was seen to be linked to transcriptional activation from binding of RARγ at the Hoxa1 3′-RARE [80]. The *myeloid ectopic viral integration site-1* (*Meis 1*) gene is involved in tissue patterning and RARγ plays a role in the epigenetic regulation of this gene. For wild-type mouse ESC lines (from blastocysts) treated with ATRA, mapping of the epigenetic signature of *Meis 1* revealed a rapid increase in the chromatin-activating H3K9/K14ac epigenetic mark at the proximal promotor and at two sites downstream of the transcriptional start site. This was not seen for RARγ^−/−^ cells treated with ATRA [81]. As considered above, *Hox* gene expression was disrupted when zebrafish embryos were treated with the RARγ agonist.

Findings from RAR/RXR Chip-seq analysis of undifferentiated F9 embryonal carcinoma cells provide support to RARs playing a role within the nucleus to regulate stem cell stemness in terms of ensuring the expression of genes for the characteristics of stem cells. The binding sites for RAR/RXR dimers coincided with the gene loci that bound stem cell pluripotency-associated transcription factors, including ESRRB, KLF4, NANOG, NR5A2, POU5f1, SOX2, and TFCP2L1. There was also a higher prevalence of the non-canonical DR0-containing retinoid response element (RARE). For differentiated cells, Sox17 binding sites were enriched in the RAR/RXR binding regions and there was a higher frequency of the canonical DR5-containing RARE. From these findings, the investigators proposed two sets of RAREs that are associated with RAR regulation of cell pluripotency and maturation, respectively. Integrated cistromic/transcriptomic analyses showed that RAR/RXR dimers had relocated when the F9 cells were treated with ATRA. The investigators concluded that global activation of RAR/RXR dimers can switch cell status from pluripotency to cell differentiation by repressing the expression of pluripotency-associated factors and cooperating with differentiation-associated factors [82].

## 5. RARγ Is a Co-Factor to Other Transcription Factors

Studies of the generation of iPSCs showed that the RARγ and Wnt/β-catenin signaling pathways are tightly connected. RARγ is the major isoform expressed in growth plate chondrocytes [83,84] and interacts with Wnt/β-catenin signaling to regulate chondrocyte function and matrix turnover. Treatment of chondrocytes that were freshly isolated from neonatal mouse epiphyseal cartilage with ATRA, in the absence and presence of Wnt3a, stimulated β-catenin signaling as revealed by TOPflash reporter assays and the accumulation of β-catenin in the nucleus. ATRA enhanced the expression of Wnt pathway components as revealed by significant increases in Wnt2a and Wnt5a proteins, the Wnt receptor Frizzled-8, and the co-receptors Lrp-5 and Lrp-6. For retinoid-free cultures, RARγ strongly inhibited Wnt/β-catenin signaling. The strong response of these cells to Wnt3a was reduced by overexpression of RARγ and to a much lesser extent by overexpression of RARα and RARβ. For cultures treated with or without Wnt3a, small interfering RNA silencing of endogenous RARγ strongly increased signaling and minimal effects were observed when RARα and RARβ were silenced. Co-immunoprecipitation studies, using COS-7 cells transfected with HA-tagged RARγ, showed that RARγ and β-catenin interacted via the N-terminal domain (AF-1) of RARγ. Inactive RARγ led to complex formation because complexes were undetectable for cells treated with ATRA. Wnt signaling allows β-catenin to accumulate in the cytoplasm and β-catenin then moves to the nucleus to interact with lymphoid enhancer factor/T cell factor, a key modulator of Wnt/β-catenin signaling. ATRA-free cultures of epiphyseal chondrocytes and two-hybrid assays were used to examine the association of β-catenin with LEF-1 whereby RARγ overexpression led to dissociation of β-catenin from LEF-1. The investigators proposed that RARγ inhibition or enhancement of Wnt/β-catenin signaling depends on the ligand status of RARγ. Unliganded RARγ would associate with β-catenin within the nucleus to prevent its interaction with lymphoid enhancer factor/T cell factor and inhibit Wnt/β-catenin signaling. In contrast, liganded RARγ would stimulate gene expression of Wnt proteins, receptors, and co-receptors to enhance Wnt/β-catenin signaling [85].

From studies of the generation of iPSCs, RARγ regulated TGFβ signaling, which is transduced by Smad3 protein. From two-hybrid and immunoprecipitation/Western studies of COS-7 cells transfected with Smad3 and RARγ, the DEF region of RARγ interacted directly with the C-terminal functional domain (MH2) of Smad3. Pan-RAR agonism (CD2043) reduced physical interactions between RARγ and Smad3, whereas pan-RAR antagonism (CD3106) potentiated [86]. Lung fibroblasts express RARγ [46]. WI-26 lung fibroblasts were transfected transiently with a Smad3-driven (CAGA)_9_-luk reporter to examine the influence of pan-RAR antagonism and pan-RAR agonism on gene transactivation. Treatment of these cells with 10 ng/mL TGFβ dramatically elevated reporter activity, and, in keeping with the above, pan-RAR agonism (CD2043 at 10^−7^ M) inhibited TGFβ-induced reporter activity and pan-RAR antagonism (CD3106 at 10^−6^ M) enhanced. Vectors were used to overexpress RARα, RARβ, and RARγ within the (CAGA)_9_-lux reporter cells and this led to increased transactivation in response to TGFβ. RARγ overexpression was the most potent RAR in enhancing the TGFβ response. When Smad3 was overexpressed instead of treating cells with TGFβ, co-expression of RARs significantly enhanced (CAGA)_9_-luk reporting, and RARγ was the most potent RAR. The overexpression experiment was repeated in lipid-free serum with identical results indicating that RARγ was likely to be inactive. When human lung fibroblasts were transfected with the reporter, Smad3, and RARγ and then treated with TGFβ, pan-RAR antagonism substantially potentiated and pan-RAR agonism abolished the potentiating effect of RARγ/Smad3 transfection on luciferase reporting. For lung fibroblasts transfected with the human type VII collagen (COL7A1) and plasminogen activator inhibitor-1 (PAI-1) gene promoters, the pan-RAR antagonist CD3106 strongly potentiated and the pan-RAR agonist CD2043 inhibited TGFβ-mediated transactivation [86]. Hence, RARγ is a co-factor to Smad3 whereby RARγ agonism inhibited TGF-induced Smad-mediated transactivation and RARγ antagonism had the opposite effect.

**Figure 2:**
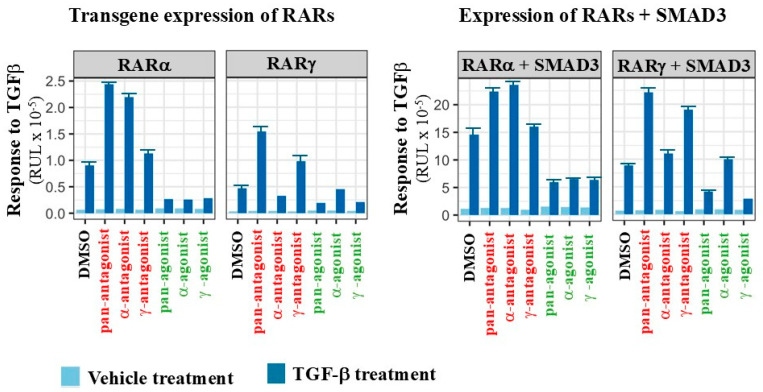
Inactive and active RARα and RARγ exert a positive and negative influence, respectively, on TGF-β and Smad3 signaling. HepG2 cells were transfected transiently (using Lipofectamine) with a Smad3-driven (CAGA)_9_-luk reporter. Vectors were used to overexpress RARα, RARβ, and RARγ and each RAR together with Smad3 (a full-length coding region) within the reporter cells. The cells were then treated with TGF-β at 10 ng/mL, for transactivation of the (CAGA)_9_-luk reporter, and with 1 × 10^−6^ M of a pan-RAR antagonist (AGN194310), an RARα antagonist (AGN196996), an RARγ antagonist (AGN205728), a pan-RAR agonist (AGN191183), an RARα agonist (AGN195183), and an RARγ agonist (AGN205327). A representative experiment for the influence of the compounds on reporter activity is shown (8 replicates for each condition). CMV-β-galactosidase was co-transfected in all experiments to monitor transfection efficiencies, and the values are β-galactosidase-normalized.

Findings for the HepG2 liver cancer cells were like those for lung fibroblasts. HepG2 cells were transfected with RARα or RARγ and the cells were then treated with selective agonists and antagonists of RARγ and RARα at 1 × 10^−6^ M (Brown unpublished, Figure 2). For HepG2 cells transfected with RARγ and then treated with 10 ng/mL TGFβ, pan-RAR agonism (AGN191183) and RARγ agonism (AGN205327) decreased TGFβ/Smad3-mediated reporting by (CAGA)_9_-luk. Conversely, pan RAR antagonism (AGN194310) and RARγ antagonism (AGN205728) enhanced reporting. Antagonism and agonism of RARα had little effect. When RARα was overexpressed, RAR agonism (AGN191183) and RARα agonism (AGN195183) decreased reporting and pan-RAR antagonism and selective RARα antagonism (AGN196996) enhanced reporting. RARγ agonism was also able to decrease reporting, perhaps in keeping with RARγ being a more potent negative modulator in lung fibroblasts and HepG2 cells constitutively expressing RARγ [87]. Similar findings were obtained when HepG2 cells were transfected to overexpress either RARγ or RARα together with Smad3 and then treated with TGFβ. From the lung fibroblast and HepG2 experimental model studies, agonizing RARα and RARγ had applied a break to the ability of cells to respond to TGFβ and antagonizing RARα and RARγ had alleviated the break. Though yet unknown, the ability of agonized RARγ to interfere with cell responsiveness to an extracellular signal may explain why active RARγ blocks stem cell development.

There are inconsistencies regarding whether active and inactive RARγ had a positive or negative effect regarding the events seen. Active RARγ had a positive effect on TGFβ signaling regarding the generation of iPSCs and negative influence on TGF-β signaling within lung fibroblasts and HepG2 cells. In keeping with this, ATRA can exert either a positive or negative effect on TGFβ signaling. Active RARγ was proposed to either modulate or have a negative influence on Wnt/β-catenin signaling within EpiSCs. Proposals to the action of RARγ within chondrocytes were that active RARγ would enhance Wnt/β-catenin signaling by stimulating the expression of Wnts and pathway proteins, and that inactive RARγ would have a negative influence on Wnt/β-catenin signaling, when Wnts were present, by preventing the interaction of β-catenin with lymphoid enhancer factor/T cell factor. The findings are for cells that are at a very different stage of development, namely iPSCs/EpiSCs and maturing chondrocytes, and the inconstancies presumably relate to active and inactive RARγ playing different roles within cells that are at a different developmental stage and in a manner that is context dependent.

RARs crosstalk with other members of the steroid/thyroid hormone nuclear receptor superfamily. Of particular interest is that RARs can form heterodimers with the thyroid hormone receptors (TRs) for triiodothyronine (T3), via a 20-amino acid region that is conserved within RAR and TR [88]. The formation of RAR/TR heterodimers was shown from overexpression and cell-free DNA-binding studies (EMSAs) and the use of luciferase reporter assays [89,90,91,92]. The three RARs formed heterodimers with TRs with equal efficiency, and the heterodimers displayed unique regulatory properties. They exhibited corepressor and coactivator properties that were distinct from those of corresponding heterodimers with RXR [92]. T3 is beneficial to the maintenance of human ESCs as seen for cells grown in a chemically defined medium. Pluripotency was maintained in medium with T3, which enhanced the maintenance of high-density cells, was compatible with long-term maintenance, and improved the cloning efficiency of cells. ESCs spontaneously differentiated when FGF2 and TGF-β were removed from the medium and the loss of pluripotency was rescued partially by T3. In this case, the addition of T3 had also decreased the growth factor dependency of human ESCs. Trophoblast development was enhanced when human ESCs were cultured with T3 and BMP4, indicating that T3 had promoted differentiation by elevating the BMP pathway [93].

## 6. RARγ Agonism or Overexpression Enhances the Proliferation of Cancer Cells

The use of ATRA, to target the RARα/PML fusion protein, in combination with arsenic trioxide, for differentiation therapy changed acute promyelocytic leukemia from a highly fatal to highly curable disease [94]. By contrast, a patient with relapsed acute myeloid leukemia was given eight days of continuous ATRA treatment, which led to rapid disease progression, and the patient died. The patients’ cells proliferated rapidly when treated in vitro with ATRA, the RARγ agonist AGN205327, and the RARα agonists AM80 and AGN195183 (all at 1 mM). Regarding RARγ, treatment of the patient’s cells in vitro with ATRA led to increased RARγ within the nucleus. The promotion of cell proliferation by RARγ appears to have, at least in part, played a role in the patient’s rapid disease progression in response to ATRA [95].

The LNCaP, PC-3, and DU145 prostate cancer cell lines are used often to investigate prostate cancer, and they express RARα and RARγ. The effect of agonizing RARγ was examined for cells that had been grown for ~10 months serum-free (ITS^+^ supplement), to avoid undue RARα activation by exogenous retinoids. Treatment of the prostate cancer cell lines with the RARγ agonist AGN205327, or a low dose of ATRA (10^−11^ to 10^−9^ M) to agonize just RARγ, enhanced cell proliferation substantially. Treatment of the cells with 10^−6^ M ATRA reduced cell proliferation. Treatment with 10^−10^ ATRA also increased the colony-forming efficiency of the cell lines. For LNCaP cells, 10^−10^ M ATRA increased the proportion of colonies that displayed morphologies consistent with either a stem-like (holoclone colonies) or early progenitor (meroclone colonies) status to 85%, and 10^−6^ M ATRA reduced the proportion of these colonies [96]. Hence, RARγ agonism enhanced the proliferation and colony-forming ability of prostate cancer cell line cells.

The predominant isoforms of RARγ within head and neck cancer are RARγ 1, 2, and 4. Transgene overexpression of each of these isoforms significantly enhanced the proliferation of human immortalized SG keratinocytes, dysplastic oral DOK keratinocytes, and the head and neck cancer cell lines FaDu, SAS, and OC3. Conversely, knockdown of RARγ abolished the proliferation of head and neck cancer cells and tumor growth in xenografted nude mice. Mechanistic studies showed that cell cycle progression was accelerated by means of RARγ 1, 2, and 4 interacting with vinexin-β and RAR ligand-dependent activation of the epidermal growth factor receptor and downstream Akt, ERK, Src, and YAP signaling pathways. They also revealed that RARγ-mediated growth promotion of head and neck cancer cells was dependent on CDK-7 phosphorylation of the AF-1 domain of RARγ. In this case, RARγ’s oncogenic role involved autocrine activation of epidermal growth factor receptor signaling to promote cell proliferation [97].

RARγ levels are high in patients’ hepatocellular cancer, cholangiocarcinoma, and colorectal cancer tissues, with RARγ residing mainly in the cell cytoplasm. Treatment of the HepG2 liver cancer cells with ATRA (at 0.1 and 1 × 10^−6^ M) enhanced cell proliferation. Silencing of RARγ expression, by using an RARγ siRNA lentiviral vector, rendered HepG2 cells sensitive to growth arrest by ATRA. Transfection of an RARγ expression vector enhanced colony formation by HepG2 cells and siRNA inhibition of RARγ impaired colony formation. Similarly, the growth of HepG2 xenografts in mice was greatly enhanced by RARγ transfection and significantly inhibited by siRNA inhibition of RARγ. From these findings, active RARγ stimulated the proliferation of HepG2 cells. Mechanistic studies showed that cytoplasmic RARγ interacted with the p85α regulatory subunit of phosphatidylinositol 3-kinase, leading to activation of Akt and NF-κB, which are critical regulators of the survival and proliferation of hepatocellular cancer cells [98]. For cholangiocarcinoma cell lines (QBC939, SK-ChA-1, and M2-ChA-1), RARγ knockdown, by using siRARγ, suppressed cell proliferation, impaired colony formation on cell culture plates, and increased sensitivity to 5-flurouracil. Knockdown led to a slower growth of xenograft tumors in nude mice, with tumors showing decreased expression of the PCNA protein, and inhibited the metastatic ability of cells as evaluated using wound healing and trans well assays. Mechanistic studies showed that RARγ knockdown inhibited activation of the Akt/NF-kB pathway and that RARγ upregulated expression of multidrug resistance 1 protein via activation of Wnt/β-catenin signaling [99]. The investigators undertook similar studies using the human colorectal cancer cell lines HT29, HCT116, and RKO, which overexpressed RARγ. Knockdown did not affect proliferation nor block cell cycle progression but did increase sensitivity to chemotherapeutics (oxaliplatin, fluorouracil, and vincristine) via downregulation of multidrug resistance 1 protein. For human colorectal cancer tissues, high expression of RARγ correlated with expression of multidrug resistance 1 protein. RARγ-mediated multidrug resistance, via upregulation of multidrug resistance 1 protein, was attributed to activation of Wnt/β-catenin signaling [100]. A recent review has highlighted the impact of retinoids in targeting multidrug resistance [101].

High-level expression of RARγ in ovarian tumors was attributed to a poor survival outcome. Knockdown of expression within the A2780 cell line, using RARγ siRNA, significantly reduced tumor growth in nude mice. The tumor cells showed decreased levels of Ki-67 and the proliferation cell nuclear antigen [102]. RARγ is overexpressed in patients’ pancreatic cancer cells and was required for cell proliferation. Reduced expression in human pancreatic cancer cell lines suppressed proliferation and tumor growth in vivo and decreased the expression of MYC and STAT3 [103]. Other workers have shown that siRNA knockdown of RARγ significantly decreased the proliferation of a pancreatic cancer PK-1 ductal adenocarcinoma and Panc-1 cell lines by arresting cells in G1 of the cell cycle without causing cell death. WikiPathway analysis revealed that many pathways related to cell cycle and DNA repair were downregulated by inhibiting RARγ. For cell lines and patient-derived organoids, blockage of RARγ signaling synergized with chemotherapy (gemcitabine) to suppress proliferation [104].

Immunohistology studies of patients’ cells have indicated a role for RARγ in disease metastasis. Overexpression in patients’ pancreatic cancer cells has been linked to aggressive disease and a poor outcome [103]. High RARγ expression was associated with lymph node metastasis of cholangiocarcinoma [99]. RARγ has been shown to be significantly elevated in high-grade prostate cancer [105] and FIGO stage III/IV of ovarian cancer [102].

From all the above studies, RARγ agonism or overexpression enhanced the proliferation of cancer cells. They also showed that active RARγ enhances the capacity of cells to give rise to colonies in tissue culture plates, mediates cell resistance to chemotherapeutics, and appears to play a role in metastasis. Agonism of RARγ enhanced the proportion of colonies that were stem-like regarding LNCaP prostate cancer cells. These findings fit well with a role for RARγ in promoting/maintaining stem cell stemness, as seen from hematopoiesis, zebrafish embryo, and iPSC studies.

RARγ is oncogenic in the above cancers by virtue of enhancing the proliferation of cells. An exception is that RARγ is a tumor suppressor for mouse keratinocytes [106]. This role was seen for skin cancer from studies of RARγ1 knockout mice and human squamous cell carcinomas biopsies and attributed to a lack of RARγ1 preventing the formation of the Riptosome RIPK1/RIPK3 death complex for necroptosis [107]. Similarly, agonism of RARγ is anti-tumorigenic in oral cavity squamous cell carcinoma. The RARγ-mediated signaling pathways within human squamous cell carcinoma cells were investigated by means of integrating genome-wide RARγ binding by Cleavage under Targets and Release Using Nuclease (CUT & RUN), chromatin histone marks, and global transcriptomics and the use of agonists and RARγ knockout cells. For these cells minus added ligand, the transcripts for NOTCH1, NOTCH3, and the NOTCH ligands JAG2 and DLL1 were reduced. Expression of these genes is associated with stratified squamous cell differentiation. The transcripts for retinaldehyde reductase DHRS3 and from genes that regulate cell identity and extracellular matrix communication were also reduced. Genes that were expressed at a higher level in knockout cells, and, therefore, repressed by RARγ included *RARG*, *PPARG*, and *RXRA*. RARγ regulation of events is, therefore, complex by virtue of its ability to regulate RXRα, which has multiple partners [108]. Why RARγ is a tumor suppressor in oral squamous cell carcinoma and oncogenic in several cancers is yet unclear. As seen for Xenopus embryo elongation, active and inactive RARγ play a role in either maintaining cells or promoting cell death pending the developmental status of cells. Perhaps the cell of origin to cancer and the developmental status of the cancer cells are important as to whether RARγ is an oncogene or a tumor suppressor.

## 7. RARγ Antagonism Kills Cancer Cells Including CSCs

ATRA-based differentiation therapy has not yielded positive clinical outcomes for other cancers [109] largely because ATRA is not very effective in arresting the growth of carcinoma cells. For example, high concentrations of ~1–10 μM were needed to arrest the growth of prostate cancer cell lines LNCaP, PC-3, and DU145 [110,111]. Therefore, ATRA has limited efficacy regarding its use to treat prostate cancer. Screening of more than 100 synthetic retinoids revealed that three RARγ agonists were the most active but, like ATRA, 1–10 μM was needed for complete growth inhibition [112]. Hence, it was interesting to explore whether antagonizing RARs would be more effective than ATRA against cancer cells.

**Figure 3:**
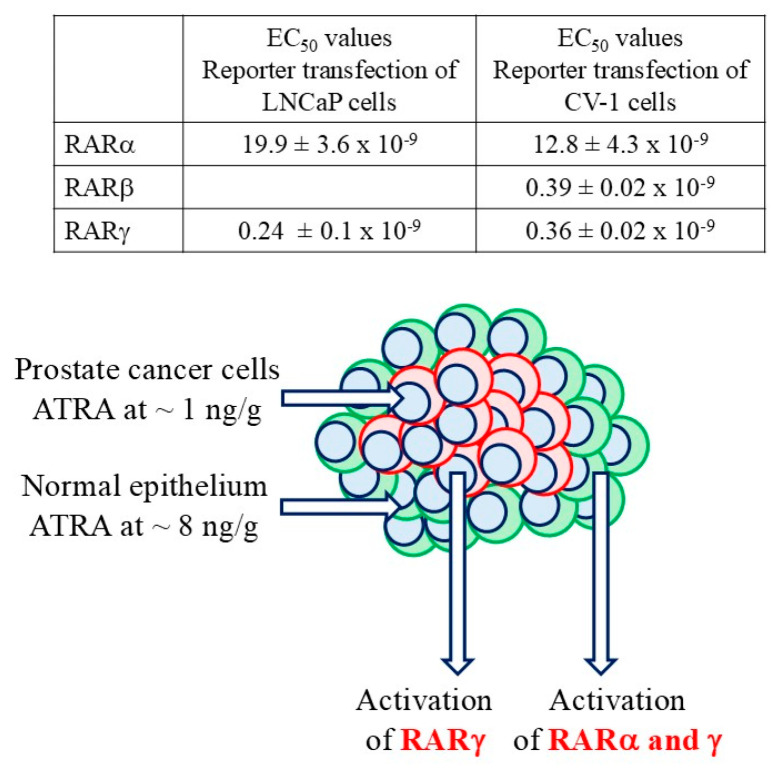
The bioavailability of ATRA to patients’ prostate cancer cells is very low. The level in patients’ cells was close to the limit of detection and 8-fold higher in adjacent normal tissue and benign prostate hyperplasia. RARγ is transactivated in LNCaP cells by sub-nM ATRA (see table) and patients’ prostate cancer cells are highly likely to be dependent on activation of RARγ for their survival.

The bioavailability of ATRA to patients’ cancer cells was an important consideration for testing RAR antagonists for activity. The level of ATRA in patients’ prostate cancer cells is very close to the limit of detection, at around 1 ng/gram tissue. The level in adjacent normal tissue and benign prostate hyperplasia is up to eight-fold higher [113]. Patients’ prostate cells are highly likely to be dependent on the activity of RARγ for survival because RARγ is transactivated within LNCaP cells by sub-mM ATRA whereas 80-fold more is required to transactivate RARα (Figure 3) [21,22]. The reduced bioavailability of ATRA is not peculiar to prostate cancer. This is also the case for pancreatic cancer [114] and impaired ATRA synthesis has been reported for human ovarian cancer cell lines [115] whereby mRNAs for the synthetic enzymes ALDH1A2, ALDH1B1, andALDH9A1 were absent [116]. The mRNAs for the ATRA synthetic enzymes ADH4, ADH1B, ADH1C, RDHL, and ALDH1A1 were low in patients’ gastric cancer cells [117], and mRNAs for ADH1B, ADH3, RDH1, and ALDH1A1 were low in non-small cell lung cancer cells [118]. ALDH1A2 expression was low in head and neck squamous cell cancer and linked to a mesenchymal-like phenotype and an unfavorable prognosis [119]. For patients’ renal cancer cells, the expression of RARγ was upregulated [120] and the levels of all-trans retinol and retinyl esters were reduced [121].

In keeping with the low bioavailability of ATRA to patients’ prostate cancer cells, potencies of the pan-RAR and RARγ antagonists were obtained for LNCaP, PC-3, and DU145 cells that had been grown long term serum-free and the use of exponentially growing cells. Primary cultures of prostate cells were also grown, as required, in the serum-free BioWhittaker Prostate Epithelium Cell Growth Medium. Therefore, the experiments examined RARγ antagonism in the absence of exogenous ATRA for RARα activation. Findings for the activities of a pan-RAR antagonist and an RARγ antagonist against prostate cancer cells are given in Table 1.

The pan-RAR antagonist was more potent than ATRA in inhibiting the proliferation of the prostate cancer cell lines (IC_50_ values of 3.5 to 5.0 × 10^−7^ M) which were arrested in GI of the cell cycle followed by necroptosis. Patients’ primary prostate cancer cells were sensitive (IC_50_ mean value of 4.6 ± 1.9 × 10^−7^ M, n = 14) and normal prostate epithelium cells and fibroblasts were less sensitive (IC_50_ values of 9.5 ± 0.8 × 10^−7^ M and 8.0 ± 0.7 × 10^−7^ M, respectively) [111]. Primary cells were obtained from patients with a Gleason grading from 5 to 10 with 9/14 samples graded as 9/10 and the cells were equally sensitive to the pan-RAR antagonist. The pan-RAR antagonist prevents colony formation by the CSC-like cell lines (IC_50_ values of 1.6–3.4 × 10^−8^ M). Plating serum-free-grown LNCaP cells in medium plus fetal bovine serum stimulated colony formation. Serum and conditioned medium from serum-free-grown LNCaP cells reversed the inhibition of colony formation by the pan-RAR antagonist, indicating that its action had interfered with an autocrine factor that stimulates colony formation [111]. The pan-RAR antagonist effectively ablated neurosphere formation by primary cells from two patients with a primitive neuroectodermal tumor and a patient with an astrocytoma [122].

Antagonism of RARγ, by using AGN205728, was sufficient to arrest the growth and drive necroptosis of the prostate cancer cell lines and prevent colony formation [96]. The RARγ antagonist was more effective against the prostate cancer cell lines (IC_50_ values of 3.5 to 5.6 × 10^−7^ M) than against the non-neoplastic human prostate epithelial RWPE-1 cells (IC_50_ values of 23 ± 4.0 × 10^−7^ M). ATRA has been shown to block the differentiation of pre-adipocyte-like cells into lipid-loaded adipocytes [123,124]. Human prostate cancer and cell lines harbor cells that are mesenchymal stem cell-like which can be induced to undergo neuroendocrine, adipogenic, or osteoblastic differentiation [125]. The RARγ agonist AGN205327 (at 10^−7^ M) inhibited adipogenic differentiation of LNCaP cells, as induced by the PPARγ agonist ciglitazone (at 10^−7^ M) [96].

The RARγ antagonists LY2955303 and MM11253 were tested against the Panc-1 and PK-1 pancreatic cancer cell lines and patient-derived organoids. The cells were arrested in G1 of the cell cycle and upregulated p21 and p27 but did not undergo apoptosis [104]. The natural flavonoid acacetin, which displaces [^3^H] ATRA from the binding site of RARγ, strongly inhibited the growth of hepatocellular cancer cells and induced apoptosis, and was active against lung, breast, and prostate cancer cells. It interfered with non-genomic actions of RARγ, which was attributed to AKT-p53 switching from being pro-survival to pro-apoptotic [87]. The pan-RAR antagonist AGN193109 was highly effective against AML in reducing LSCs. An aggressive subtype of AMK is characterized by overexpression of *Ecotropic virus integration site 1* (*Evi1*). In an *MLL-AF9*-driven murine model of AML and from in vitro studies, *Evi1* increased the abundance and activity of LSCs and reduced the maturation of leukemic cells. These effects were augmented by ATRA, in an *Evi1*-dependent manner, and treatment of the cells with the pan-RAR antagonist led to effects that were opposite to those of ATRA. In vivo, the pan-RAR antagonist reduced LSC abundance and delayed leukemogenesis. The pan-RAR antagonist had counteracted the effects of *Evi1* on AML stemness. There is the prospect of using the pan-RAR antagonist to treat *EVII^high^* AML [126].

673A, DIMATE, DEAB, NCT-501, disulfiram, silybin, and solomargin blocked RAR signaling by inhibiting the enzymes to ATRA biosynthesis. 673A induced necroptosis of human CD133^+^ ovarian cancer stem-like cells [127]. DIMATE induced apoptosis of H1650 and H1975 non-small cell lung cancer cell line cells and was effective against xenografts [128]. DEAB, NCT-501, and disulfiram preferentially killed ALDH^high^ uterine endothelial cancer cells and reduced spheroid cell formation by patients’ uterine endothelial cancer stem cells. Tumorigenesis of spheroid cells in vivo was suppressed by disulfiram [129]. Silybin downregulated RARγ expression and reduced the growth of tumors when ALDH1A1^+^ prostate cancer DU145 cells were transplanted into nude mice [130]. The viability of human ovarian cancer cell line cells was decreased by solomargine, which also inhibited the growth of the cell line A2780CP70 in mouse xenografts [131]. Findings from all the above studies support the use of either RAR antagonists or agents that block ATRA synthesis to kill CSCs.

## 8. The Role of RARγ Is Multifaceted

RARγ mRNA is expressed mainly by primitive cells and liganded RARγ blocked stem cell development, as seen from studies of different models. Activity of RARγ was not essential to maintaining HSCs, which were present to just a lesser extent in the bone marrow of RARγ knockout mice. Moreover, the maintenance of stem cells is also tightly controlled by extracellular signals and communication between stem cells and their niche environment. Therefore, RARγ modulates the propensities of stem cells to either maintain stemness or develop as governed by many external influences. RARγ is expressed alongside RARα within stem cells. The level of ATRA governs activation of either just RARγ or both RARγ and RARα, by virtue of their differential transactivation, and, therefore, RARγ can play a dominant role.

Transactivation of RARγ by sub-nM ATRA is relevant to stem cells. ESCs appear to be unable to synthesize ATRA because of a lack of the specific cell surface receptor stimulated by retinoic acid 6 for all-trans retinol and the ATRA synthetic enzymes, including the alcohol dehydrogenases Adh4, and Adh1 and retinaldehyde dehydrogenase RALDH2 [132,133]. mRNAs for RARγ and RARβ were readily detected from a global gene expression analysis of feeder-free undifferentiated mouse ESCs (a clone of D3 cells) and the mRNAs for retinal dehydrogenases, for the conversion of retinol to ATRA, were at very low levels. Even so, the cytoplasm of undifferentiated mouse ESCs contained ~1 nM ATRA, as measured by Liquid Chromatography-Mass Spectrometry, whereby ESCs can take up ATRA from fetal calf serum. A nM level of ATRA, as sufficient to transactivate RARγ, regulated the expression of responsive genes because treatment of ESCs with the RAR inverse agonist BMS493 (to increase repression) decreased the basal expression of the *RARb* and *Hox1* genes and increased the expression of the known ATRA-repressed genes *Foxd3* and *Otx2*. In contrast, treatment of ESC embryoid bodies with 5 μM ATRA was detrimental to stemness and set differentiation within these cells to initiate distinct genetic programs [134]. During embryogenesis, we might presume that stem cells rely on a supply of ATRA from specialized cells that synthesize ATRA. The levels are tightly controlled spatially and temporally during embryogenesis to ensure correct tissue patterning during development [135] and ATRA proximity was needed for RARγ2 to become active during Xenopus embryo elongation. Sufficient ATRA for activation of RARγ and, in turn, RARγ-mediated regulation of gene expression and/or inhibition of extracellular signaling events, may ensure that stem cells maintain their stemness.

**Figure 4:**
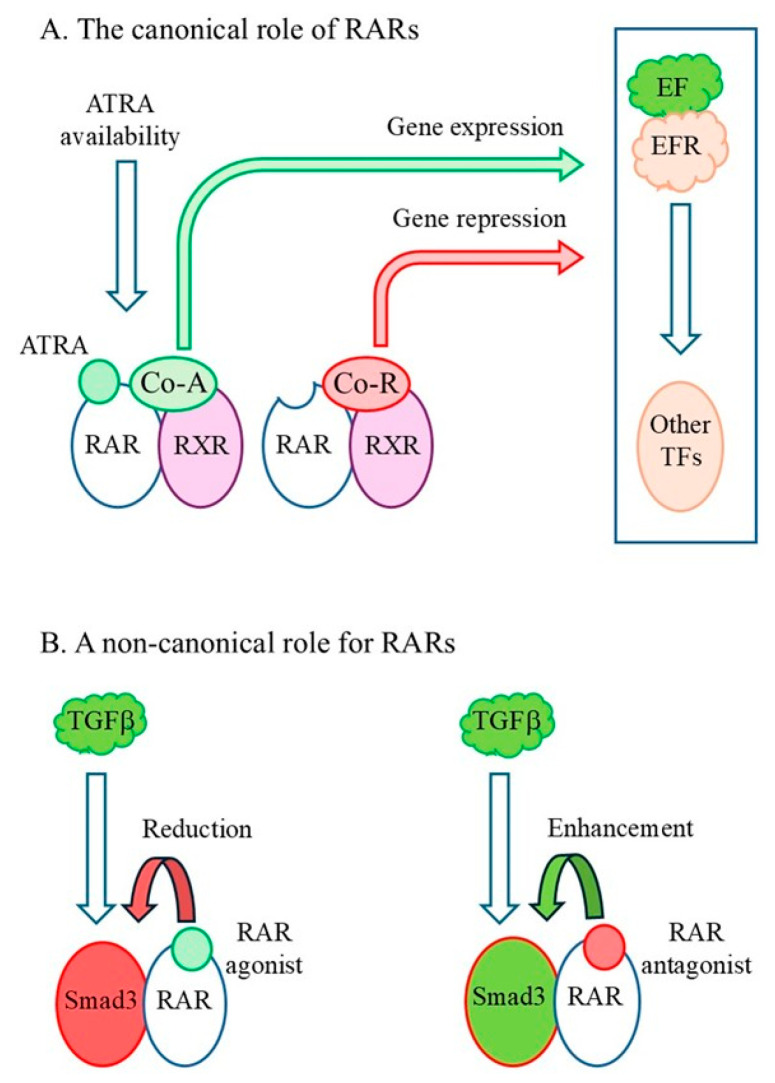
The regulatory roles of RARs. (**A**). For ATRA target genes, RARs act as a transcriptional activator when ligand is bound and as a repressor when ligand is absent. The regulated genes encode molecules that play key roles in controlling cell behavior. (**B**). A non-canonical role for RARs. RARs can either apply a break to or enhance events that are actioned by other transcription factors in response to an extracellular signal. Active RARγ and RARα applied a break to such events and inactive RARs enhanced TGFβ-driven Smad3 transactivation. RARγ was more potent than RARα. EF, extracellular factor; EFR, extracellular factor receptor, TF, transcription factor; Co-A, co-activator; Co-R, co-repressor; RXR, retinoid X receptor.

The RAR signaling network is very extensive, as shown from an integrated genomics analysis of ATRA-induced differentiation of F9 ESCs whereby RARγ sits within a complex array of molecules and pathway interactions [136]. Moreover, how RARs change cell behavior is multifaceted. The canonical role is to activate the transcription of target genes when the ligand is bound and repress gene expression when the ligand is absent (Figure 4A). Active and inactive RARγ both play a biological role and the molecules that are regulated are prime components to pathways that control stem cell behavior, including Wnts and Wnt receptors [85], NOTCH1, NOTCH3, the NOTCH ligands JAG2 and DLL1 [108], Smads [61], *Hox* genes [58], and molecules relating to autocrine growth factor signaling [97] (Figure 4A). RARγ also acts as a co-factor to other transcription factors [86]. Both RARα and RARγ integrated extracellular signals that are important to control stem and progenitor cell behavior by either enhancing or applying a break to events that are mediated by other transcription factors, as seen from studies of Smad3 transactivation within lung fibroblasts and HepG2 cells in response to TGFβ (see Figure 4B). Presumably, this ensures a tight control on whether cells do or do not change their behavior in response to an external signal. In addition to the presence of RARγ within the nucleus, RARγ localizes to the cytoplasm due to its unique N-terminal A/B domain. RARγ was cytoplasmic in confluent cells, when cells were treated with growth factors or released from serum starvation, and when ectopically overexpressed [137]. Perhaps RARγ shuttles between the two compartments to play different roles. For HepG2 and cholangiocarcinoma cells, activity of cytoplasmic RARγ led to activation of the Akt/NF-kB pathway [98,99]. The various prescribed roles for RARγ lead towards the view that, by different means, it modulates extracellular signal-provoked events that change the behavior of normal and cancer stem cells. Perhaps the ability of agonized RARγ to interfere with the capacity of cells to respond to extracellular signals provides a rationale to why agonized RARγ blocked stem cell development and facilitated ground-state pluripotency. The cellular means to these outcomes might be somewhat similar.

## 9. Concluding Remarks

RAR-mediated gene expression and suppression lead to cells changing their nature and behavior with RARγ and RARα playing different roles during hematopoiesis. There are many unknowns to how their canonical roles might coordinate the propensities of stem cells to maintain stemness or veer away from this status. They include whether RARα and RARγ compete for binding to RXRs, bind to different RAREs, and whether there is dynamic exchange at RAREs, and one RAR can override another one. RARγ forms dimers with other steroid hormone nuclear receptors and acts as a co-factor to other transcription factors. The breadth of these interactions is yet unclear. RARγ influences chromatin marks and chromatin modeling whereby widespread changes to the epigenome are important to the positive and negative influences of RARγ. RARγ also modulates intracellular signaling as seen for the phosphatidlyinositol-3-kinase/Akt/NF-κB pathway. A recent paper that mathematically modeled the epigenome regarding hematopoiesis highlighted overlapping transcription programs and transcription factor competition whereby enhancers compete for epigenetic readers. The identity and stability of cell states were modeled in terms of “attractor patterns” [138]. In so much as RARγ interacts with many molecules, an understanding of RARγ “attractor patterns” may highlight how RARγ governs cell behavior. Also, there is the need to study events at a single-cell level to resolve the various roles of RARγ.

For cancer cells, overexpression and agonism of RARγ enhanced cell proliferation with RARγ antagonism leading to cell death, including of CSCs and particularly in the absence of RARα activation. There is the possible use of pan-RAR and RARγ antagonists to kill CSCs to treat aggressive cancers and/or disease relapse. This would include in combination with conventional chemotherapeutics because, for example, the use of the RARγ antagonist together with docetaxel was more effective against prostate cancer cell lines than each agent alone [96]. For prostate cancer, there is still the need to look at antagonist efficacy in xenograft mouse models. Also, antagonizing RARγ might kill normal stem cells and there is limited information to the extent this might be the case. Surprisingly, no adverse effects were seen other than an inhibition of spermatogenesis, which was reversible, when mice and rats were given a substantial dose of the pan-RAR antagonist BMS-18945 for some time [139,140]. 673A, as used to inhibit ALDH1A and ATRA production, showed selectivity for ovarian CSCs exhibiting little toxicity to mesenchymal stem cells and non-malignant breast cells [127]. DIMATE, as used to inhibit ALDH1 and ALDH3, eradicated LSCs and spared normal hematopoietic progenitors [141]. Perhaps normal stem cells are more resilient to antagonism of RARs than CSCs. Other areas that are yet to be explored regarding the therapeutic use of antagonists include their influence on the stromal environment of cells and immune cells.

## Figures and Tables

**Table 1:** RAR antagonists are potent inhibitors of the growth of prostate cancer cells and prevent colony formation by cell line cells. For the colony-forming assay, cells were plated in tissue culture dishes, treated with agents, and colonies were assessed on day 12. The IC_50_ values for cell cultures were measured at day 5 by using the Vialight™ HS High Sensitivity Cell Proliferation/Cytotoxicity assay (Lonza, UK). All values are the mean ± standard errors of means from at least 3 replicate experiments. Similar values were obtained from counting harvested viable cells on day 5. * The value shown for the activity of the pan-RAR antagonist against primary patients’ prostate cancer cells is the mean obtained for 14 patient samples.

Cells	Pan-RAR Antagonist (AGN194310)	RARγ Antagonist (AGN205728)	ATRA
**For CSC-like colony-forming cells (IC_50_ values, relative to control)**			
LNCaP	1.6 ± 0.5 × 10^−8^ M	4.2 ± 0.1 × 10^−8^ M	34.4 ± 3.2 × 10^−8^ M
PC3	1.8 ± 0.6 × 10^−8^ M	4.7 ± 0.2 × 10^−8^ M	41.9 ± 12 × 10^−8^ M
DU145	3.4 ± 0.7 × 10^−8^ M		40.2 ± 7.4 × 10^−8^ M
**For cell line cultures (IC_50_ values, viable cells)**			
LNCaP	3.9 ± 0.2 × 10^−7^ M	4.5 ± 0.2 × 10^−7^ M	>2 × 10^−6^ M
PC3	3.5 ± 0.4 × 10^−7^ M	4.7 ± 0.2 × 10^−7^ M	>2 × 10^−6^ M
DU145	5.0 ± 0.4 × 10^−7^ M	5.6 ± 0.2 × 10^−7^ M	>2 × 10^−6^ M
Non-malignant RWPE-1		23 ± 4.0 × 10^−7^ M	
**For primary cell cultures (IC_50_ values, viable cells)**			
Prostate cancer cells	4.6 ± 1.9 × 10^−7^ M *	3.0 × 10^−7^ M	>2 × 10^−6^ M
Non-malignant epithelium	9.5 ± 0.8 × 10^−7^ M	7.2 ± 0.5 × 10^−7^ M	
Normal fibroblasts	8.0 ± 0.7 × 10^−7^ M		

## Data Availability

The original contributions presented in this study are included in the article. Further inquiries can be directed to the corresponding author.
